# Development and validation of a nomogram for predicting severe respiratory syncytial virus-associated bronchiolitis

**DOI:** 10.1186/s12879-023-08179-y

**Published:** 2023-04-18

**Authors:** Jisi Yan, LiHua Zhao, Tongqiang Zhang, Yupeng Wei, Detong Guo, Wei Guo, Jun Zheng, Yongsheng Xu

**Affiliations:** 1 Department of Respiratory Medicine, Tianjin University Children’s Hospital (Tianjin Children’s Hospital), 238 Longyan Road, Beichen District, Tianjin, 300000 China; 2 Clinical School of Pediatrics, Tianjin Medical University, Tianjin, China; 3 Department of Neonatology, Tianjin Central Hospital of Gynecology Obstetrics, 156 Nankai SAN Lu, Nankai District, Tianjin, 300100 China

**Keywords:** Severe bronchiolitis, Respiratory syncytial virus, Nomogram, Independent predictor, Infants and young children

## Abstract

**Background:**

Respiratory syncytial virus (RSV) is the most common cause of bronchiolitis and is related to the severity of the disease. This study aimed to develop and validate a nomogram for predicting severe bronchiolitis in infants and young children with RSV infection.

**Methods:**

A total of 325 children with RSV-associated bronchiolitis were enrolled, including 125 severe cases and 200 mild cases. A prediction model was built on 227 cases and validated on 98 cases, which were divided by random sampling in R software. Relevant clinical, laboratory and imaging data were collected. Multivariate logistic regression models were used to determine optimal predictors and to construct nomograms. The performance of the nomogram was evaluated by the area under the characteristic curve (AUC), calibration ability and decision curve analysis (DCA).

**Results:**

There were 137 (60.4%) mild and 90 (39.6%) severe RSV-associated bronchiolitis cases in the training group (*n* = 227) and 63 (64.3%) mild and 35 (35.7%) severe cases in the validation group (*n* = 98). Multivariate logistic regression analysis identified 5 variables as significant predictive factors to construct the nomogram for predicting severe RSV-associated bronchiolitis, including preterm birth (OR = 3.80; 95% CI, 1.39–10.39; *P* = 0.009), weight at admission (OR = 0.76; 95% CI, 0.63–0.91; *P* = 0.003), breathing rate (OR = 1.11; 95% CI, 1.05–1.18; *P* = 0.001), lymphocyte percentage (OR = 0.97; 95% CI, 0.95–0.99; *P* = 0.001) and outpatient use of glucocorticoids (OR = 2.27; 95% CI, 1.05–4.9; *P* = 0.038). The AUC value of the nomogram was 0.784 (95% CI, 0.722–0.846) in the training set and 0.832 (95% CI, 0.741–0.923) in the validation set, which showed a good fit. The calibration plot and Hosmer‒Lemeshow test indicated that the predicted probability had good consistency with the actual probability both in the training group (*P* = 0.817) and validation group (*P* = 0.290). The DCA curve shows that the nomogram has good clinical value.

**Conclusion:**

A nomogram for predicting severe RSV-associated bronchiolitis in the early clinical stage was established and validated, which can help physicians identify severe RSV-associated bronchiolitis and then choose reasonable treatment.

## Background

Bronchiolitis is a lower respiratory tract disease usually caused by respiratory virus infection in infants and young children [1, 2]. It imposes a significant medical and economic burden on societies. In some developed countries, including the United States, 108,000 individuals were hospitalised for bronchiolitis in 2016, costing 734 million dollars [3]. The treatment burden for bronchiolitis is increasing annually. An analysis of bronchiolitis hospitalisations in England between 1979 and 2011 revealed that the annual hospitalisation rate for bronchiolitis in 2011 was 46.1 per 1,000 infants younger than 1 year (95% CI, 45.6–46.6), which poses a seven-fold increase over the 32-year period [4]. Furthermore, bronchiolitis has consumed significant resources of paediatric intensive care units (ICUs), where it accounted for 9,628 (27.6%) cases of 34,829 ICU admissions in Australia and New Zealand between 2002 and 2014, resulting in direct costs exceeding $30 million per year [5]. Furthermore, bronchiolitis is the leading cause of infant hospitalisation in the developing countries, including China [6, 7]**.** Children with bronchiolitis accounted for 12.25–21.24% of the total number of hospitalised patients with respiratory tract infections at the First Hospital of Jilin University in China between January 2005 and December 2014, with an average hospital stay duration of 7.78 ± 3.47 days [8]. Furthermore, infants with bronchiolitis, especially those with severe cases, are more likely to develop cardiovascular and liver damage [9].

Respiratory syncytial virus (RSV) is the most common cause of bronchiolitis, accounting for 41–83% of all patients with bronchiolitis [2]. Moreover, RSV infection is associated with increased hospitalisation rate and bronchiolitis severity [10–12]. In 2015, there were an estimated 33.1 million cases of RSV-associated acute lower respiratory tract infections in children under 5 years, most of which were mild, while approximately 3.2 million cases required hospitalisation and 59,600 died in hospitals worldwide [13]. Most severe cases and deaths occur in children under 2 years, and most deaths occur in developing countries [14]. Therefore, it is important to identify and treat RSV-associated bronchiolitis in children at risk of developing severe diseases at an early stage. Many studies have investigated the risk factors and predictors of severe RSV-associated bronchiolitis, and the commonly recognised risk factors are preterm birth, young age, congenital heart diseases, bronchopulmonary dysplasia, and immunodeficiency diseases [15, 16]. The other risk factors are controversial, such as male sex, low weight at admission, history of tobacco exposure, lack of breastfeeding (< 1 month), anaemia, coinfections, and high viral load. [14, 17–21]. However, only few studies have reported on developing a simple and accurate model to predict severe RSV-associated bronchiolitis in the early stages. Therefore, this study aimed to develop and validate a nomogram for predicting the risk of severe RSV-associated bronchiolitis among infants and young children and provide a theoretical basis for clinical identification.

## Method

### Selection of the study sample

A retrospective study was conducted in 325 patients with RSV-associated bronchiolitis who were hospitalized at Tianjin Children's Hospital in China from January 2018 to December 2021. This study was approved by the ethics committee of the Tianjin Children’s Hospital and conducted in accordance with the Declaration of Helsinki guidelines.

The following three inclusion criteria were met by all the children enrolled in this study: (1) age < 2 years, which was consistent with the diagnostic criteria of the American Academy of Pediatrics (AAP) Guidelines in 2014 and the National Institute for Health and Care Excellence (NICE) Guidelines in 2021 for the Clinical Diagnosis of bronchiolitis [22, 23]; (2) showing symptoms and signs of lower respiratory diseases at admission which was consistent with the clinical diagnostic criteria of bronchiolitis, including cough; wheezing; nasal flaring; inspiratory rales; expiratory wheezing on auscultation; and intercostal, subcostal, or supraclavicular retractions [15, 23]; and (3) a positive test for nucleic acid of RSV in the nasopharyngeal secretions. The specimens of the children were collected at admission, and a viral nucleic acid extraction kit (Hubei Langde Medical Technology Limited Company) was used to extract the viral RNA by magnetic bead extraction. Polymerase chain reaction (PCR) amplification was then performed. The following factors were used to identify positive experimental results: S-shaped amplification curve in the FAM channel and a CT value ≤ 38.

Severe bronchiolitis was diagnosed according to the expert consensus on the diagnosis, treatment and prevention of bronchiolitis (2014 edition) [24], any of the following criteria was met: (1) feeding drops to more than half of normal or refusal to eat; (2) respiratory rates > 70 breaths per minute; (3) three concave signs (intercostal, subcostal, and supraclavicular retractions); (4) nasal flaring or moaning; (5) blood oxygen saturation < 88%; or (6) extreme restlessness, lethargy, or coma.

The exclusion criteria included any of the following: (1) a course of illness for more than 2 weeks when admitted to the hospital; (2) nosocomial infection; (3) automatic discharge or death during hospitalization; (4) co-bacterial infection; or (5) incomplete medical records.

### Collection of clinical data

All the patients’ medical data were collected at hospital admission and discharge, including demographic information, medical history, physical examination, etiological examination, laboratory data, imaging features and clinical management, as follows: (1) demographic information: age, gender, region (rural/urban), weight at admission, preterm birth, mode of delivery (natural birth/cesarean section), history of birth asphyxia, birth weight, feeding mode (breast/formula feeding), underlying diseases (including hemodynamically significant heart disease, chronic lung disease, immunodeficiency disease, etc.), history of wheezing, history of eczema; (2) medical history and physical examination: fever, cough and wheezing days before admission, use of antibiotics and systemic corticosteroids before admission, breath rate, three concave signs (intercostal, subcostal, or supraclavicular retractions), abnormal lung auscultation (including moist rales and/or stridor); (3) etiological examination: the nasopharyngeal secretions of the children were collected on admission, and a viral nucleic acid extraction kit produced by Hubei Langde Medical Technology Limited Company was used to extract viral RNA. Then, PCR amplification was performed to detect 7 respiratory pathogen nucleic acids (including respiratory syncytial virus, adenovirus, influenza virus A, influenza virus B, parainfluenza virus tpye1-3). Two sputum samples and one blood sample were collected simultaneously for Gram staining and culturing in growth medium to exclude bacterial infection and galactomannan by antigen assay to exclude fungal infection; (4) lung X-ray examination (no obvious abnormality/visible infiltration, emphysema, atelectasis and pleural effusion); (5) laboratory data: white blood cell (WBC), neutrophil ratio (N%), lymphocyte ratio (L%), eosinophil ratio (E%), hemoglobin (Hb), C-reactive protein (CRP), procalcitonin (PCT), interleukin-6 (IL-6), lactic acid (La), aspartate aminotransferase (AST), alanine aminotransferase (ALT), creatine kinase (CK), creatine kinase isomer-MB (CK-MB), lactic dehydrogenase (LDH), immunoglobulin E (IgE); (6) hospital course: hospitalization days.

### Statistical methods

#### Variable selection and model construction

We used R software with the “sample ()” function to randomly divide the data of the 325 patients into a training group (70%, 227 patients) and a validation group (30%, 98 patients). All 227 patients in the training dataset were analysed for variable selection and risk prediction. Variables significant (*P* < 0.1) at univariate analysis were included in the multivariable model.

The selected factors were used to develop the risk prediction model for severe RSV-associated bronchiolitis and were presented as a nomogram using R software with the “rms” packages. The nomogram proportionally converts each variable to a scale of 0–100 points based on the regression coefficient. The absolute value of the variable with the highest β coefficient in the multivariable logistic regression was assigned 100 points. The value of each selected variable was marked to obtain the corresponding points. The total points, based on the sum of the points for each predictor in this nomogram, were associated with the risk of severe RSV infection.

#### Validation of the nomogram

The performance of the prediction model was evaluated based on its discrimination ability, calibration ability and clinical value. The discrimination ability was evaluated through the area under the receiver operator characteristic curve (AUC), and the calibration plot accompanied by the Hosmer‒Lemeshow test was applied to assess the calibration ability. The model was validated using the bootstrap method with 1000 resamples to quantify any overfitting. Decision curve analysis (DCA) was applied to evaluate the clinical utility of the nomogram based on its net benefits at different threshold probabilities.

In addition, for better clinical application of the nomogram, the total scores of each patient were calculated based on the model. The ROC analysis was used to explore the optimal cutoff value for predicting severe RSV-associated bronchiolitis, and the critical value with maximum Youden index (sensitivity + specificity-1) was determined. Accuracy of the optimal cutoff value was assessed by the sensitivity and specificity.

#### Statistical analysis

Continuous variables were expressed as the mean ± standard deviation (SD) or median values (interquartile range) and assessed by independent group t tests or Mann‒Whitney U tests. Categorical variables were expressed as percentages (%) and assessed by Chi-squared tests or Fisher’s exact test. Statistical analysis was carried out using SPSS 26.0, and R software (version 4.0.5, http://www.r-project.org) was used to perform all the graphics based on the R packages “foreign”, “rms”, “ggplot2”, “pROC” and “glmnet”. *P* < 0.05 was considered statistically significant, and factors with *P* < 0.1 in the univariate analysis were included in multivariate logistic regression analysis.

## Result

### Patient characteristics

A total of 325 children with RSV infection met the inclusion criteria, among which 206 (63.4%) were male, and 125 cases were diagnosed as severe RSV infection. The data were randomly divided into a training group (*n* = 227) and a validation group (*n* = 98). There were 90 (39.6%) and 35 (35.7%) patients with severe RSV infection in the training and validation groups, respectively. The baseline clinical data and proportion of severe RSV infection were similar between the training and validation groups, except for serum AST level and length of wheezing (Table 1).

**Table 1:** The variables of training and validation cohort

	Patients (*n* = 325)	Training group (*n* = 227)	Validation group (*n* = 98)	*P*
Age, months^b^	5(2–10)	5(2–10)	5(3–9.3)	0.936
Sex, male/female	206/119	146/81	60/38	0.595
weight at admission, kg^b^	7.8(6.3–9.5)	7.7(6.2–9.6)	7.8(6.5–9.4)	0.836
Region, rural/urban	110/215	79/148	31/67	0.580
preterm birth, n(%)	36(11.1%)	27(11.9%)	9(9.2%)	0.475
cesarean section, n(%)	171(52.6%)	121(53.3%)	50(51%)	0.705
history of birth asphyxia, n(%)	6(1.8%)	5(2.2%)	1(1%)	0.467
birth weight, kg^b^	3.3(3.0–3.6)	3.3(3.0–3.6)	3.3(2.9–3.7)	0.664
non-breastfeeding, n(%)	173(53.2%)	124(54.6%)	49(50%)	0.443
underlying diseases, n(%)	16(4.9%)	12(5.3%)	4(4.1%)	0.645
history of wheezing, n(%)	20(6.2%)	16(7.0%)	4(4.1%)	0.307
history of eczema, n(%)	143(44%)	106(46.7%)	37(37.8%)	0.136
Fever, n(%)	178(54.8%)	128(56.4%)	50(51%)	0.372
Length of cough, days^b^	4(3–6)	4(3–6)	4(3–8)	0.681
Length of wheezing, days^b^	1(0–3)	1(0–3)	2(0–4)	0.017
Outpatient use of antibiotics, n(%)	247(76%)	168(74%)	79(80.6%)	0.201
Outpatient use of glucocorticoids, n(%)	86(26.5%)	57(25.1%)	29(29.6%)	0.401
Breath rate^b^	45(40–51)	45(40–51)	44(40–51.5)	0.845
Three concave signs, n(%)	98(30.2%)	66(29.1%)	32(32.7%)	0.579
Abnormal lung auscultation, n(%)	322(99.1%)	224(98.7%)	98(100%)	0.253
Abnormal radiological findings, n(%)	198(60.9%)	141(62.1%)	57(58.2%)	0.503
Length of stay, days^b^	6(5–7)	6(5–7)	5(4–7)	0.303
WBC, × 10^9^/L^a^	8.8 ± 3.3	8.6 ± 3.3	9.3 ± 3.2	0.105
N%^a^	32.2 ± 15.4	33.1 ± 15.6	29.9 ± 14.8	0.078
L%^a^	57.2 ± 15.1	56.1 ± 15.1	59.5 ± 14.8	0.063
E%^b^	0.0(0.0–2.0)	0.0(0.0–2.0)	0.0(0.0–2.0)	0.540
Hb, g/L^b^	115(106–121)	114(105–121)	111(104.5–119)	0.166
CRP, mg/L^b^	2.5(2.5–2.7)	2.5(2.5–3.0)	2.5(2.5–2.5)	0.384
PCT, ng/ml^b^	0.07(0.05–0.10)	0.07(0.05–0.10)	0.07(0.05–0.10)	0.265
IL-6, pg/ml^b^	7.6(4.4–13.4)	7.7(4.4–12.4)	7.1(4.2–15.7)	0.985
La, mol/L^b^	3.2(2.6–4.0)	3.2(2.6–3.9)	3.3(2.5–4.3)	0.786
AST, U/L^b^	44(36–59)	43.0(34.0–58)	47(38.8–63)	0.038
ALT, U/L^b^	22(17.0–34.0)	22.0(16–32)	24.5(18.0–37.3)	0.057
CK, U/L^b^	103.0(74–146)	104(78–146)	100.5(69.5–146)	0.543
CKMB, U^b^/L	12(7–23)	12(7.0–23)	12(7.0–24.3)	0.663
LDH, U/L^b^	398((340.5–493.5)	392(337–482)	415.5(346.3–515)	0.079
IgE, IU/L^b^	12.9(3.8–40.6)	11.5(3.3–38.7)	12.4(4.5–47.4)	0.851

*Abbreviations*: *WBC* White blood cell, *N* Peripheral neutrophils, *L* Peripheral lymphocytes, *E* Peripheral eosinophils, *Hb* Hemoglobin, *CRP* C-reactive protein, *PCT* Procalcitonin, *IL-6* Interleukin (IL)-6, *La* Lactic acid, *AST* Aspartate aminotransferase, *ALT* Alanine aminotransferase, *CK* Creatine kinase, *CKMB* Creatine kinase isomer-MB, *LDH* Lactic dehydrogenase, *IgE* Immunoglobulin E

^a^Mean ± SD

^b^Median (IQR)

### Predictors of severe RSV infection

In the training group, children with severe RSV infection had younger median age (3 m vs 6 m, *P* < 0.001), lower median weight at admission (6.5 kg vs 8.4 kg, *P* < 0.001), higher proportion of country (44.4% vs 28.5%, *P* = 0.013), higher preterm birth rate (17.8% vs 8%, *P* = 0.026), higher cesarean section rate (62.2% vs 47.4%, *P* = 0.029), higher incidence of underlying disease (8.9% vs 2.9%, *P* = 0.049), faster median respiratory rate (51b vs 40b, *P* < 0.001), higher three concave signs rate (73.3% vs 0%, *P* < 0.001), longer median duration of hospital (7d vs 5d, *P* < 0.001), lower mean level of Hb (111 g/L vs 115 g/L, *P* = 0.024), median IgE (8.7U/L vs 14.8U/L, *P* = 0.027), higher median level of CK (110.5U/L vs 97U/L, *P* = 0.011), CKMB (16.5U/L vs 11U/L, *P* = 0.022) and LDH (415U/L vs 380U/L, *P* = 0.042) compared with those mild RSV infection (Table 2).

**Table 2:** The clinical, laboratory and radiological features of RSV-associated bronchiolitis in the training group

	training group (*n* = 227)	mild bronchiolitis (*n* = 137)	severe bronchiolitis (*n* = 90)	*P*
Age, months^b^	5(2–10)	6(3–12)	3(1.8–7.0)	0.000
Sex, male/female	146/81	84/53	62/28	0.244
weight at admission, kg^b^	7.7(6.2–9.6)	8.4(7.1–9.8)	6.5(5.2–8.6)	0.000
Region, rural/urban	79/148	39/98	40/50	0.013
preterm birth, n(%)	27(11.9%)	11(8%)	16(17.8%)	0.026
cesarean section, n(%)	121(53.3%)	65(47.4%)	56(62.2%)	0.029
history of birth asphyxia, n(%)	5(2.2%)	1(0.7%)	4(4.4%)	0.062
birth weight, kg^b^	3.3(3.0–3.6)	3.3(3.0–3.5)	3.3(2.9–3.6)	0.672
non-breastfeeding, n(%)	124(54.6%)	76(55.5%)	48(53.5%)	0.751
underlying diseases, n(%)	12(5.3%)	4(2.9%)	8(8.9%)	0.049
history of wheezing, n(%)	16(7.0%)	11(8%)	5(5.6%)	0.476
history of eczema, n(%)	106(46.7%)	67(48.9%)	39(43.3%)	0.410
Fever, n(%)	128(56.4%)	89(65%)	39(43.3%)	0.001
Length of cough, days^b^	4(3–6)	4(3–6)	4(3–6)	0.687
Length of wheezing, days^b^	1(0–3)	1(0–3)	1(0–3)	0.05
Outpatient use of antibiotics, n(%)	168(74%)	101(73.7%)	67(74.4%)	0.903
Outpatient use of glucocorticoids, n(%)	57(25.1%)	29(21.2%)	28(31.1%)	0.091
Breath rate^b^	45(40–51)	40(38–45)	51(46–56)	0.000
Three concave signs, n(%)	66(29.1%)	0(0%)	66(73.3%)	0.000
Abnormal lung auscultation, n(%)	224(98.7%)	135(98.5%)	89(98.9%)	0.822
Abnormal radiological findings, n(%)	141(62.1%)	83(60.6%)	58(64.4%)	0.558
Length of stay, days^b^	6(5–7)	5(4–6)	7(6–8)	0.000
WBC, × 10^9^/L^a^	8.6 ± 3.3	8.6 ± 3.4	8.7 ± 3.2	0.819
N%^a^	33.1 ± 15.6	32.1 ± 15.4	34.8 ± 15.7	0.206
L%^a^	56.1 ± 15.1	57.6 ± 14.6	53.9 ± 15.7	0.073
E%^b^	0.0(0.0–2.0)	0.0(0.0–2.0)	0.0(0.0–2.0)	0.607
Hb, g/L^b^	114(105–121)	115(107–123)	111(104–119.3)	0.024
CRP, mg/L^b^	2.5(2.5–3.0)	2.5(2.5–2.8)	2.5(2.5–3.7)	0.614
PCT, ng/ml^b^	0.07(0.05–0.10)	0.07(0.06–0.10)	0.08(0.05–0.11)	0.661
IL-6, pg/ml^b^	7.7(4.4–12.4)	7.7(3.8–14.4)	7.6(4.7–13.2)	0.712
La, mol/L^b^	3.2(2.6–3.9)	3.2(2.6–4.0)	3.2(2.7–3.9)	0.741
AST, U/L^b^	43.0(34.0–58)	45(35–56.5)	41(33.8–60.3)	0.403
ALT, U/L^b^	22.0(16–32)	21.0(15–31.5)	22(16.8–33.3)	0.200
CK, U/L^b^	104(78–146)	97(67–139)	110.5(87.5–157.8)	0.011
CKMB, U/L^b^	12(7.0–23)	11(7.0–20.5)	16.5(9.0–30.0)	0.022
LDH, U/L^b^	392(337–482)	380(334.5–447)	415.0(338.8–538.5)	0.042
IgE, IU/L^b^	11.5(3.3–38.7)	14.8(4.2–47.7)	8.7(2.8–25.3)	0.027

*Abbreviations*: *WBC* White blood cell, *N* Peripheral neutrophils, *L* Peripheral lymphocytes, *E* Peripheral eosinophils, *Hb* Hemoglobin, *CRP* C-reactive protein, *PCT* Procalcitonin, *IL-6* Interleukin (IL)-6, *La* Lactic acid, *AST* Aspartate aminotransferase, *ALT* Alanine aminotransferase, *CK* Creatine kinase, *CKMB* Creatine kinase isomer-MB, *LDH* Lactic dehydrogenase, *IgE* Immunoglobulin E

^a^Mean ± SD

^b^Median (IQR)

Univariate logistic regression showed that age (OR = 0.90; 95% CI, 0.85‒0.95; *P* < 0.001), weight at admission (OR = 0.74; 95% CI, 0.65‒0.85; *P* < 0.001), children from rural areas (OR = 2.01; 95% CI, 1.15‒3.51; *P* = 0.014), preterm birth (OR = 2.48 95% CI, 1.09‒5.62; *P* = 0.03), cesarean section (OR = 1.82; 95% CI, 1.06‒3.14; *P* = 0.030), fever history (OR = 0.41; 95% CI, 0.24‒0.71; *P* = 0.001), breathing rate (OR = 1.16; 95% CI, 1.11‒1.21; *P* < 0.001), level of CKMB (OR = 1.02; 95% CI, 1.00‒1.03; *P* = 0.039) and LDH (OR = 1.002; 95% CI, 1.000‒1.004; *P* = 0.036) were significant predictors of severe RSV-associated bronchiolitis (Table 3).

**Table 3:** Univariate and multivariate logistic regression for predicting severe RSV-associated bronchiolitis in the training group

Variables	Univariate analysis OR (95% CI)	*P* value	Multivariate analysis OR (95% CI)	*P* value
Age, months	0.895(0.847–0.946)	0.000	0.995(0.894–1.106)	NS
weight at admission	0.742(0.649–0.850)	0.000	0.758(0.631–0.910)	0.003
Region	2.010(1.151–3.510)	0.014	1.292(0.640–2.610)	NS
preterm birth	2.477(1.091–5.621)	0.03	3.802(1.392–10.388)	0.009
cesarean section	1.824(1.061–3.138)	0.03	1.665(0.852–3.252)	NS
history of birth asphyxia	6.326(0.695–57.540)	0.102		
underlying diseases	3.244(0.947–11.113)	0.061	2.110(0.480–9.286)	NS
Fever	0.412(0.239–0.711)	0.001	0.503(0.242–1.044)	NS
Length of wheezing	1.072(0.962–1.195)	0.205		
Breath rate	1.160(1.109–1.213)	0.000	1.111(1.047–1.179)	0.001
Outpatient use of glucocorticoids	1.682(0.918–3.083)	0.093	2.267(1.048–4.9)	0.038
L%	0.984(0.967–1.002)	0.074	0.968(0.946–0.991)	0.001
Hb	0.982(0.961–1.002)	0.083	0.997(0.968–1.026)	NS
CK	1.001(1.000–1.001)	0.083	1.001(1.000–1.002)	NS
CKMB	1.017(1.001–1.033)	0.039	0.985(0.962–1.008)	NS
LDH	1.002(1.000–1.004)	0.036	1.002(1.000–1.005)	NS
IgE	1.000(0.998–1.001)	0.454		

*Abbreviations*: *L* Peripheral lymphocytes, *Hb* Hemoglobin, *CK* Creatine kinase, *CKMB* Creatine kinase isomer-MB, *LDH* Lactic dehydrogenase, *IgE* Immunoglobulin E, *NS* No significance, *CI* Confidence interval

On multivariate regression analysis, weight at admission (OR = 0.76; 95% CI, 0.63‒0.91; *P* = 0.003), preterm birth (OR = 3.80; 95% CI, 1.39‒10.39; *P* = 0.009), faster breathing rate (OR = 1.11; 95% CI, 1.05‒1.18; *P* = 0.001), outpatient use of glucocorticoids (OR = 2.27; 95% CI, 1.05‒4.9; *P* = 0.038) and lymphocyte percentage (OR = 0.97; 95% CI, 0.95‒0.99; *P* = 0.001) were independent predictors for severe RSV-associated bronchiolitis (Table 3).

### Development and validation of a severe RSV infection-predicted nomogram

**Fig. 1:**
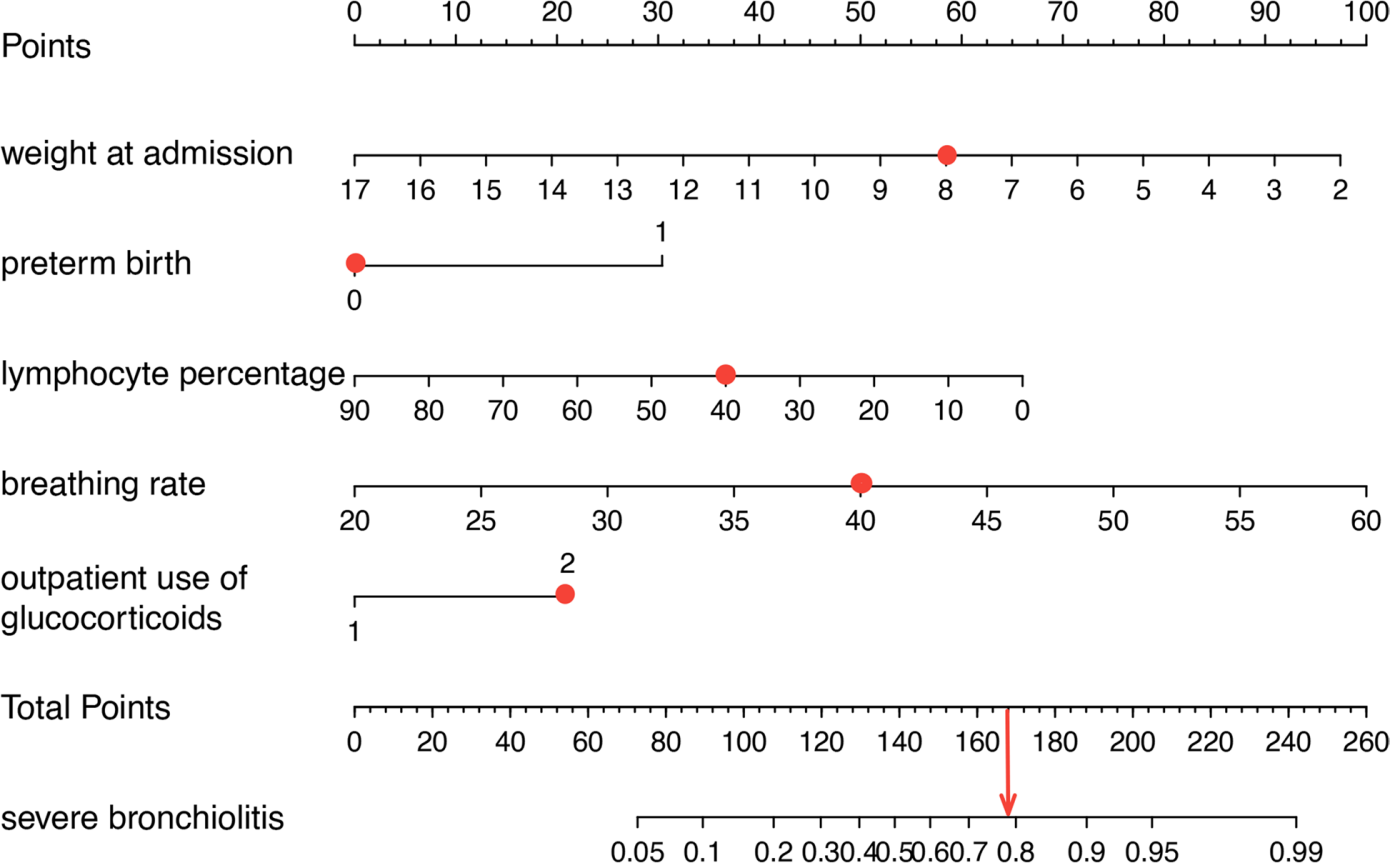
Nomogram to predict severe bronchiolitis among children with RSV infection was constructed based on 5 independent predictors. Mark the value of these included factors on the corresponding axis. Draw a vertical line from the value to the top lines and get corresponding points. Then, sum the points from each variable value. Locate the sum on the total points scale and project it vertically on the bottom axis to obtain a severe bronchiolitis risk

The 5 variables identified by multivariate regression analysis were applied to establish the risk model and presented with a nomogram (Fig. 1): weight at admission, presence of preterm birth, percentage of lymphocytes, breathing rate and outpatient use of glucocorticoids. Total points based on the sum of the points for each predictor in this nomogram were associated with the risk of severe RSV infection.

**Fig. 2:**
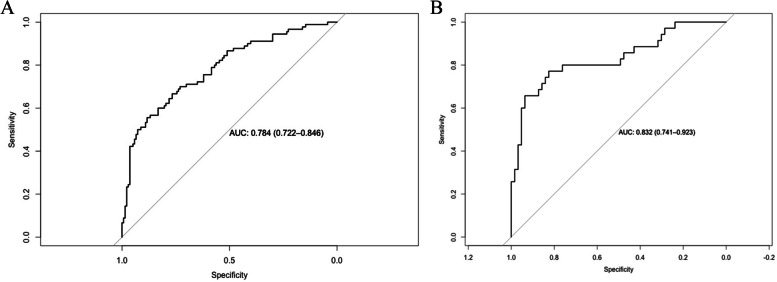
The ROC curves of the nomogran from the training dataset (**A**) and the validation dataset (**B**). ROC: receiver operating characteristics

**Fig. 3:**
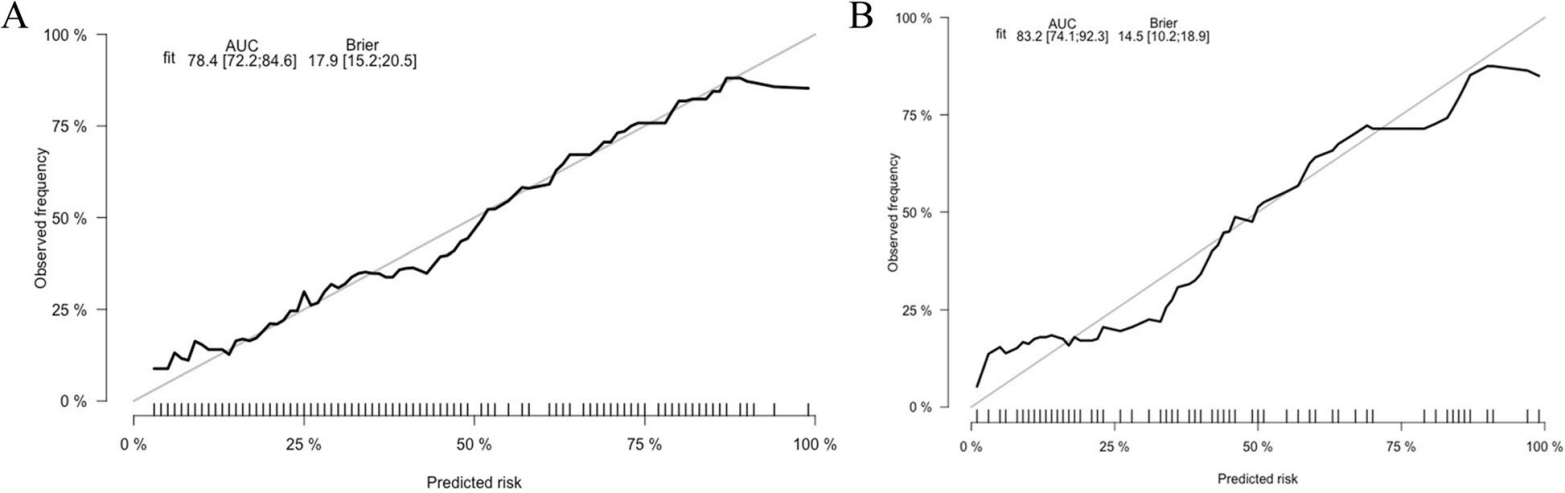
Calibration plot of severe bronchiolitis risk nomogram in the development cohort (**A**) and validation cohort (**B**). The ideal outcome (dashed line), the observed outcome (fine dashed line), and the bias-corrected outcome (solid line) are depicted

By internal bootstrap validation with 1000 resamples, the mean AUC of the nomogram based on the training group was 0.784 (95% CI, 0.722‒0.846) (Fig. 2A), with good discrimination ability for predicting severe RSV infection. Furthermore, the calibration plot (Fig. 3A) and Hosmer‒Lemeshow test (*P* = 0.817) of the prediction model showed good consistency between the predicted probability and actual probability.

**Fig. 4:**
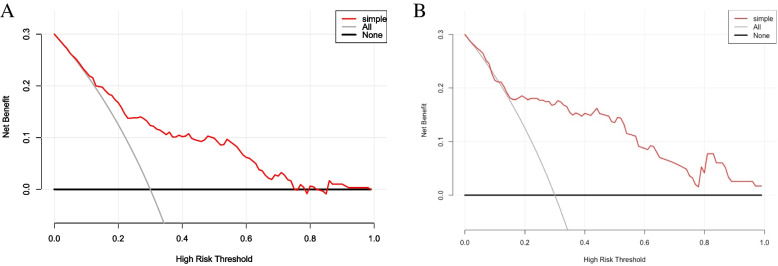
Decision curve analysis for the severe bronchiolitis risk nomogram. They-axsi measuered the net benefit. The black solid line represented the assumption that all patients had mild bronchiolitis. The gray solid line represented the assumption that all patients had severe bronchiolitis. The red solid line represented the risk nomogram. **A** From the training dataset and (**B**) from the validation dataset

The accuracy of the nomogram in the validation dataset was similar to that of the training group, with an AUC value of 0.832 (95% CI, 0.741‒0.923) (Fig. 2B). The calibration plot (Fig. 3B) and Hosmer‒Lemeshow test (*P* = 0.290) showed that the prediction model fit well in the validation dataset. The DCA curve showed obvious net benefits of the predictive nomogram and was significantly higher than those of the two extreme cases (Fig. 4).

The ROC analysis identified that the optimal cutoff value with maximum Youden index was 126. The sensitivity and specificity in recognizing severe RSV-associated bronchiolitis was 72.7%, 81.6% in the training dataset and 70.5%, 82.1% in the validation dataset, respectively.

## Discussion

In this study, we developed and validated a nomogram for predicting severe RSV-associated bronchiolitis. A total of 37 candidate variables were considered in the development of the nomogram. Moreover, five significant predictors were identified using multivariate logistic regression method, namely preterm birth, weight at admission, breathing rate, lymphocyte ratio, and outpatient administration of glucocorticoids. A nomogram model, with good discrimination, calibration, and clinical value, was developed and validated based on these five variables for predicting severe RSV-associated bronchiolitis. As an example to better explain the nomogram model, if a patient of RSV infection is weight at admission of 8 kg (58 points), preterm birth of no (0 point), lymphocyte percentage of 40% (37 points), breathing rate of 40 (50 points) and outpatient use of glucocorticoids is yes (23 points), the probability of severe RSV infection is estimated to be 78%. The predictive model is easy to operate and helpful for the early identification and timely treatment of high-risk infants and young children with bronchiolitis.

Preterm birth is reported to be a significant risk factor for severe RSV-associated bronchiolitis [15, 25–27], which is consistent with our findings that preterm-born children were 3.8 times more likely to develop severe RSV infection In a historical cohort study of preterm-born infants (≤ 32 gestational weeks) [25], the hospitalisation rate for RSV-related infection from birth to 1-year corrected age was approximately 11.2%. Furthermore, the hospitalisation rates increased with the decrease in the gestational age (13.9% for preterm-born infants ≤ 26 gestational weeks vs. 4.4% for those born at 30–32 gestational weeks). A tertiary medical centre study [27] reported that between 2001 and 2019, a total of 3,311 infants were hospitalised for bronchiolitis, with a hospital length of stay of 2.5–3 days, and the gestational age was associated with the length of stay (R = -0.12; *P* = 0.000). Additionally, our study showed that preterm birth was closely associated with the severity of RSV-associated bronchiolitis. A possible explanation may be attributed to the fact that preterm birth interrupts normal lung development in utero, resulting in significant changes in the pulmonary physiology and function. In preterm babies, the true alveolar structure is not completely formed, their surfactant and cortisol systems are immature, and their foetal lung is not ready for gas exchange, making them more likely to develop severe respiratory diseases [28–31].

Our findings revealed that children who received systemic glucocorticoids in an outpatient setting were 2.27 times more likely to develop severe RSV-associated bronchiolitis than those who did not. However, due to the controversial efficacy of inhaled or systemic glucocorticoids in acute bronchiolitis, the current expert consensus in China on the diagnosis and treatment of bronchiolitis recommends that [24] glucocorticoids are not recommended for routine use. Previous studies reported that the current evidence does not support an association between undergoing glucocorticoid treatment and the severity of bronchiolitis [32, 33]. Ricardo et al. [33] conducted a meta-analysis of 17 randomised controlled trials (2,596 children with acute bronchiolitis) and reported that glucocorticoids did not significantly reduce the hospitalisation rates compared to the placebo (RR = 0.92; 95% CI, 0.78–1.08; *P* = 0.30). However, some studies have reported that early administration of oral dexamethasone in the treatment of patients with acute bronchiolitis can reduce the hospitalisation rates (19% vs. 44% for dexamethasone group vs. placebo group, respectively) in children aged < 24 months (*P* = 0.039) [34]. The efficacy of glucocorticoids may increase in children with severe bronchiolitis. Furthermore, paediatricians tend to prescribe glucocorticoids to reduce inflammation and relieve asthma in children with obvious wheezing, which in turn increases the administration rates of glucocorticoids [35]. This may explain the association between glucocorticoid administration and severe bronchiolitis in our study.

In severe bronchiolitis, airway resistance shows a significant increase, with obstruction of the small airways due to mucus blockage and airway wall oedema as well as bronchospasm in the relatively narrow airways in infants, resulting in an increased breathing rate and work of breathing [35]. Kohei et al. [36] reported that respiratory rates > 70 breaths per minute on the day of admission were associated with subsequent transfer to the ICU or use of mechanical ventilation (OR = 4.64; 95% CI, 2.86–7.53; P < 0.001) in a multicentre cohort study of 2,104 children hospitalised for bronchiolitis. Serge et al. [37] conducted a cohort study of 378 children with bronchiolitis, 117 (31%) of whom were hospitalised. They reported that a respiratory rate > 45 breaths per minute (sensitivity, 68%; specificity, 82%; RR, 4.57), could predict the severity of lung disease and the need for hospitalisation. The results of these two studies are consistent with our findings, where the increase in the breathing rate can predict the severity of bronchiolitis (OR = 1.11; 95% CI, 1.05–1.18; *P* = 0.001), and our study provides data that support this argument from the perspective of the prediction model.

In our findings, the white blood cell count was not significantly different (*P* = 0.819) between the RSV-mild group (8.6 ± 3.4 × 10^9^/L) and the RSV-severe group (8.7 ± 3.2 × 10^9^/L). However, the results of multivariate regression analysis showed that a decreased lymphocyte ratio (53.9 ± 15.7) was an independent risk factor for severe RSV infection (OR = 0.97; 95% CI, 0.95–0.991; *P* = 0.001). Paola et al. [18] reported that a lymphocyte count < 3,200/μL (OR = 5.23; 95% CI 1.40(19.47); *P* = 0.014) was an independent risk factor for severe bronchiolitis, which was consistent with our results. This may indicate an insufficient adaptive immune response in the infants and young children in the severe group. A previous study [38], reported that the pathogenesis of viral lower respiratory tract infection is mainly related to the failure to form an adaptive cytotoxic T lymphocyte response and insufficient secretion of IL-2, IL-4, IFN-γ by T lymphocytes.

Malcolm et al. [19] reported that a higher weight (kg) on admission in children with bronchiolitis reduced the risk of requiring mechanical ventilation (adjusted OR = 0.51; 95% CI, 0.40–0.65; *P* < 0.001). Our study also showed that a higher admission weight could lower the odds of severe cases (OR = 0.76; 95% CI, 0.63–0.91; *P* = 0.003). The weight of infants and young children shows a specific exponential relationship with their chronological age according to the WHO Child Growth Criteria [39]. Furthermore, lung development is affected by the weight of the children. With the increase in weight in infants and young children, the size of the lungs, the number of alveoli, and the maturity of the lungs increase [29, 31, 40].

In addition, Paola et al. [18] and Jonathan et al. [41] reported that age is a significant predictor of severe bronchiolitis; however, this was not the case in our study (OR = 0.99; 95% CI, 0.90–1.11; *P* > 0.05). We believe this might be related to the large age span of our study (0–2 years), while the age of the samples studied by Paola et al*.* [18] was within the range of 0–1 year. It may also have something to do with our small sample size (*n* = 325) and different races [14, 41]. Our study participants were Chinese, while the study populations of Paola et al*.* [18] and Jonathan et al. [41] were Europeans and Americans, respectively. The region had no effect in our multivariate logistic analysis (OR = 1.29; 95% CI, 0.64–2.61; *P* > 0.05), while in the study by Marcello et al. [42], the living environment and conditions were independent risk factors for severe bronchiolitis. This may be related to the different national and socioeconomic conditions in the regions where our study was conducted.

This study has some limitations. Environmental factors, including air pollution (primary traffic pollution, ozone, and PM 2.5) and tobacco smoke exposure, were not collected in the medical records. Previous studies reported that these factors increase the severity of RSV infections [43, 44]. In terms of aetiology, our study examined the nucleic acids of seven common respiratory pathogens (including RSV, adenovirus, influenza viruses A and B, and parainfluenza virus types 1–3); however, some studies suggested that coinfection with other viruses, including rhinovirus and human bocavirus, increases the hospitalisation rate of RSV-associated bronchiolitis [11, 41]. Regarding applicability, our study participants were patients aged < 2 years, and the results are applicable to children in this age group, but not in children with bronchiolitis in other age groups. Furthermore, our study was a single-centre study, and all research data were collected from one medical centre, which may limit its application in other fields and necessitates the development of a multicentre study.

In conclusion, we developed and validated a nomogram for predicting severe RSV-associated bronchiolitis in the early clinical stage, which is easy and convenient for the physicians to use.

## Data Availability

The datasets used and analysed during the current study are available from the corresponding author on reasonable request.

## Abbreviations

RSV: Respiratory syncytial virus
AUC: Receiver operator characteristic Curve
DCA: Decision curve analysis
ICU: Intensive care unit
PCR: Polymerase chain reaction
WBC: White blood cell
N: Peripheral neutrophils
L: Peripheral lymphocytes
E: Peripheral eosinophils
Hb: Hemoglobin
CRP: C-reactive protein
PCT: Procalcitonin
IL-6: Interleukin (IL)-6
La: Lactic acid
AST: Aspartate aminotransferase
ALT: Alanine aminotransferase
CK: Creatine kinase
CKMB: Creatine kinase isomer-MB
LDH: Lactic dehydrogenase
IgE: Immunoglobulin E
SD: Standard deviation
LOS: Length of stay
ETS: Tobacco smoke exposure
HBoV: Human bocavirus
